# Dissection of the long-range projections of specific neurons at the synaptic level in the whole mouse brain

**DOI:** 10.1073/pnas.2202536119

**Published:** 2022-09-26

**Authors:** Jiaojiao Tian, Miao Ren, Peilin Zhao, Shukang Luo, Yingying Chen, Xiaofeng Xu, Tao Jiang, Qingtao Sun, Anan Li, Hui Gong, Xiangning Li, Qingming Luo

**Affiliations:** ^a^Britton Chance Center and MoE Key Laboratory for Biomedical Photonics, Wuhan National Laboratory for Optoelectronics, Huazhong University of Science and Technology, Wuhan 430074, China;; ^b^Key Laboratory of Biomedical Engineering of Hainan Province, School of Biomedical Engineering, Hainan University, Haikou 570228, China;; ^c^Research Unit of Multimodal Cross Scale Neural Signal Detection and Imaging, Chinese Academy of Medical Sciences, HUST-Suzhou Institute for Brainmatics, JITRI, Suzhou 215125, China

**Keywords:** synapse, synaptophysin, whole brain, basal forebrain, Alzheimer's disease

## Abstract

Through synaptic connections, long-range circuits transmit information among neurons and connect different brain regions to form functional motifs and execute specific functions. Tracing the synaptic distribution of specific neurons requires submicron-level resolution information. However, it is a great challenge to map the synaptic terminals completely because these fine structures span multiple regions, even in the whole brain. Here, we develop a pipeline including viral tracing, sample embedding, fluorescent micro-optical sectional tomography, and big data processing. We mapped the whole-brain distribution and architecture of long projections of the parvalbumin neurons in the basal forebrain at the synaptic level. These neurons send massive projections to multiple downstream regions with subregional preference. With three-dimensional reconstruction in the targeted areas, we found that synaptic degeneration was inconsistent with the accumulation of amyloid-β plaques but was preferred in memory-related circuits, such as hippocampal formation and thalamus, but not in most hypothalamic nuclei in 8-month-old mice with five familial Alzheimer’s disease mutations. Our pipeline provides a platform for generating a whole-brain atlas of cell-type-specific synaptic terminals in the physiological and pathological brain, which can provide an important resource for the study of the organizational logic of specific neural circuits and the circuitry changes in pathological conditions.

## Significance

It is a huge challenge to dissect the projection patterns of specific neurons with synaptic information in the whole brain. Here, we establish a pipeline for studying long-range projection patterns at the synaptic level and acquired whole-brain information of parvalbumin neurons in the basal forebrain. The synaptic terminals of parvalbumin neurons are widely distributed in the limbic system, including the hippocampus, thalamus, and mammillary body. In the mouse model of Alzheimer’s disease, we found that synaptic degeneration of parvalbumin neurons occurred in memory-related regions, which are inconsistent with amyloid-β plaque distribution. These works provided anatomical information for understanding how basal forebrain regulates downstream regions and the tools to investigate synaptic connections of specific neurons in the whole brain under physiological and pathological conditions.

Neural circuits are composed of different types of neurons through long-range and local connections with synapses, which act as communication nodes (1). Synaptic connections are the key determinant of neural information transmission from upstream neurons to downstream neurons. The synaptic distribution in the targeted areas, including the location and number of presynaptic components, has been linked to the physiological activity of neural circuits (2–4). Changes in synaptic organization are associated with dysfunction in neurological and psychiatric diseases, such as synaptic degeneration in Alzheimer’s disease (AD) and Parkinson’s disease (1, 5), while preventing or reversing the process of synaptic loss can improve cognition in animal models (6, 7). Therefore, quantitative investigation of the synaptic organization pattern under physiological and pathological conditions is essential for understanding the functional organization of the neuronal circuits and the pathological mechanism of psychiatric diseases. Although neuronal connections, such as the complete connectome of *Caenorhabditis elegans*, can be reconstructed at the single-synapse level with electron microscopy (8), it is labor intensive and unsuitable for large-volume samples, such as the whole mouse brain. Moreover, identifying the synapses of specific neuron types in electron microscopy datasets remains a major challenge. Many optical, physiological, and histological techniques have been developed to investigate synaptic organization in specific circuits in vivo and in vitro (4, 9–11). However, these studies at the synaptic level mainly focus on small-scale samples or local areas of circuits.

Long-range projections connect the neurons in different areas, modulate information processing, and synchronize activities across multiple brain regions. Previous studies on long-range circuits have mainly focused on physiological and anatomical characterization via magnetic resonance imaging and electroencephalography. The resolution of these techniques is not suitable for the detection of synaptic structures in the whole brain. Although there were some results for the distribution of synapses in multiple brain areas even in the whole brain, the data were from only a few interesting slices (12, 13) and lacked the continuous information for circuit dissection. Mapping the axonal and synaptic terminals of specific neuron types in the whole brain requires imaging with submicron resolution and high throughput. This requirement brings with it the challenge of the dissection of the synaptic connectome, from sample processing to maintaining the structure and fluorescent signals, to imaging in large-scale volumes with high resolution and big data processing.

Herein, to continuously map the projection pattern with synaptic information in the whole brain, we combined recent advanced techniques to label and image neuronal circuits. We labeled the presynaptic elements of the specific neurons by viral tracing (14) in the basal forebrain (BF), which participates in complex functions through abundant connections with the hippocampus, cortex, and thalamus (15, 16). Then, we modified the sample processing to keep the fluorescence from being quenched and acquired the whole-brain distribution of synaptic terminals with the fluorescence micro-optical sectioning tomography (fMOST) system (17, 18). With these continuous datasets, we reconstructed and quantified the three-dimensional (3D) distribution of the synapse in different downstream regions and investigated the synaptic changes in mice with five familial AD mutations (5×FAD). In general, the pipeline can be useful for the study of the synaptic distribution and characteristics of specific neurons and facilitate the understanding of neural circuit organization.

## Results

### Pipeline for Acquiring the Synaptic Terminals of Specific Types of Neurons.

**Fig. 1.**
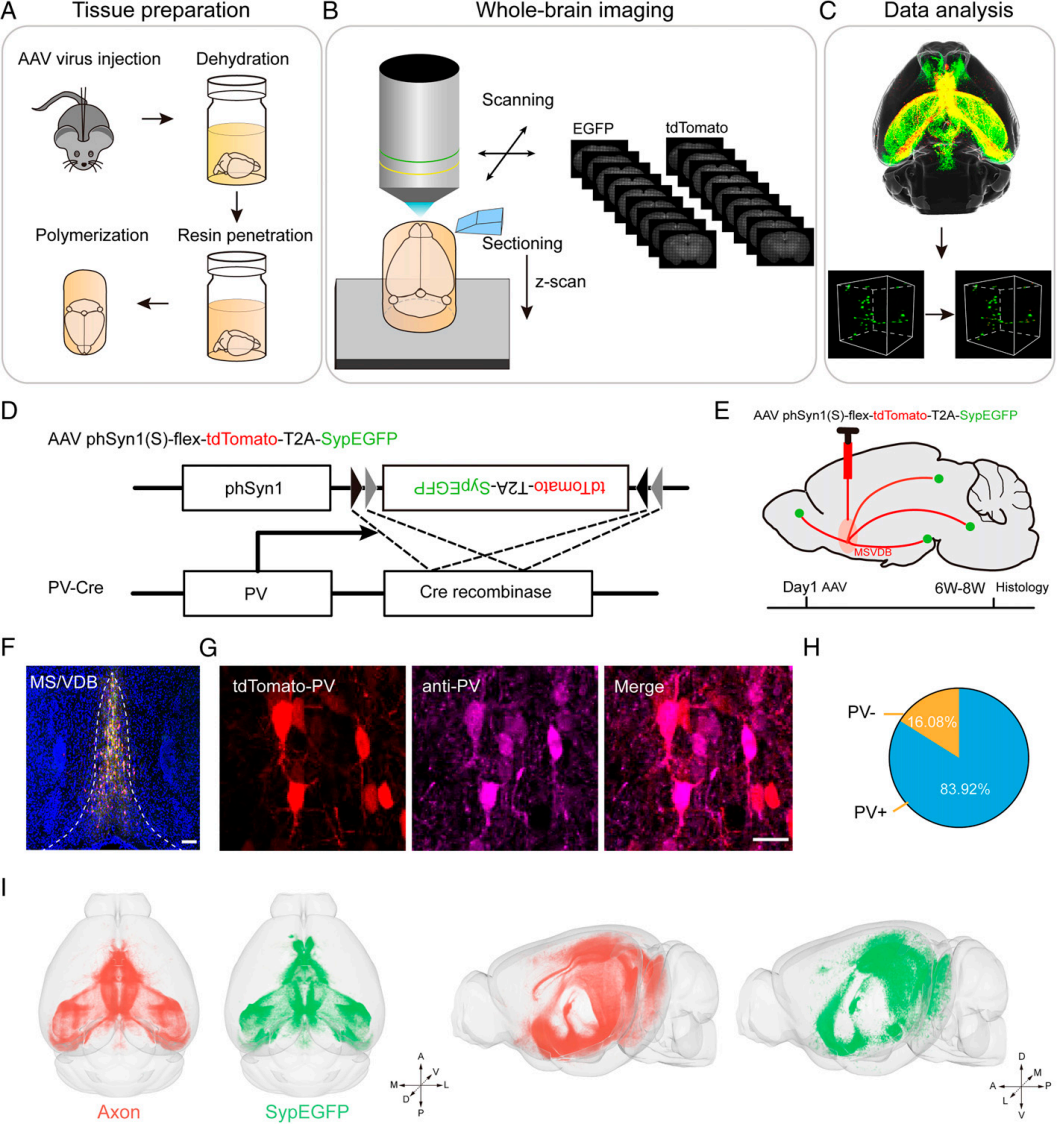
Pipeline for the construction of the axonal projections and synaptic terminals of PV^+^ neurons in the MS/VDB. (*A*) Virus injection and sample treatment process of the embedding method. (*B*) Whole-brain imaging with the fMOST system at a resolution of 0.2 × 0.2 × 1 μm and data preprocessing. (*C*) The 3D dataset was reconstructed, analyzed, and registered to the Allen CCFv3 Brain Atlas. (*D*) Diagram of the virus for axonal projection and synaptic connection. (*E*) The procedure of virus expression in the MS/VDB. The red signal denotes tdTomato-labeled fibers projected from the MS/VDB brain area. The green signal denotes EGFP-labeled synaptophysin. (*F*) Fluorescence images of MS/VDB in the PV mouse brain. (Scale bar: 100 μm.) (*G* and *H*) Immunohistochemical staining and quantification of PV^+^ neurons in the MS/VDB. (Scale bar: 20 μm.) (*I*) Horizontal and sagittal views of the whole brain. The red signals represent axon distribution. The green signals represent synaptic distribution. A, anterior; D, dorsal; L, lateral; M, medial; P, posterior; V, ventral.

To map the distribution of presynaptic elements of specific neuron types in the whole brain, we combined viral tracing, fMOST imaging, and data processing (Fig. 1 *A*–*C*). As an example, we investigated the synaptic terminal distribution of the parvalbumin-positive (PV^+^) neurons, which represent ∼7% of GABA (γ-aminobutyric acid)-containing neurons in the BF (19) and show high activity in terms of cognitive activity, spatial working memory, and neural network regulation (20–22). To label these specific projections, we injected the AAV2/9-hSyn-flex-tdTomato-T2A-synaptophysin-EGFP-WPRE-pA virus into the medial septum nucleus (MS) and diagonal band nucleus (VDB) of PV-ires-Cre transgenic mice (Fig. 1 *D* and *E*).

The virus can label the soma and axonal projections with tdTomato, while green fluorescent protein (GFP) was fused with synaptophysin, a presynaptic integral membrane protein located in the presynaptic vesicles (23). After 6–8 wk of expression, the mice were euthanized for the subsequent analyses. To clarify the specificity of virus infection, we checked the virus expression at the injection site. Most of the virus-infected somas (98.73 ± 0.11%, *n* = 5 mice) were restricted to the MS/VDB area (Fig. 1*F* and *SI Appendix*, Fig. S1 *A* and *B*). To evaluate the specificity of virus expression, we determined the neuron type of fluorescent protein-labeled neurons via immunochemical staining. Most tdTomato-labeled soma (83.93 ± 2.33%) were in PV-positive neurons (Fig. 1 *G* and *H*), while no neurons were labeled in C57BL/6J mice with the same tracing method (*SI Appendix*, Fig. S1*C*). These results indicated the specificity of the virus we used.

To verify that the synaptophysin-EGFP (enhanced green fluorescent protein) fusion protein (SypEGFP) was located in the presynaptic structure, we performed immunostaining with antibodies against GFP and synaptophysin (*SI Appendix*, Figs. S1*D* and S2*A*). The results showed that 98.65 ± 0.53% of synaptophysin expressed with EGFP (*SI Appendix*, Fig. S1*E*) and 97.37 ± 0.67% of EGFP signals colocalized with synaptophysin (*SI Appendix*, Fig. S2*C*). The quantitative results revealed that the expression of SypEGFP had high specificity (*SI Appendix*, Fig. S2 *A* and *B*). In addition, we immunostained with antibodies for endogenous synaptic markers, such as synapsin, SV2, SNAP-25, and syntaxin, to further answer this question (*SI Appendix*, Fig. S2 *D* and *E*). Synapsin and SV2 are vesicular proteins, and SNAP-25 and syntaxin are proteins on the presynaptic membrane. The results showed that many SypEGFP puncta were colocalized with synapsin, SV2, SNAP-25, and syntaxin. These results indicated that the presynaptic structure expressed the SypEGFP fusion proteins. The validity of the detection and quantification of synaptic parameters by confocal microscope data were established by the high correlation of data from structure illumination microscopy (SIM) (*SI Appendix*, Fig. S2 *F–H*).

The GFP-positive presynaptic elements were ∼1 μm or even smaller. To investigate the connections at the synaptic level, we needed to acquire the structural information with submicron voxel resolution. In addition, we needed to obtain the long-range projection over many brain regions. With fMOST (18), we imaged the block face, cut the imaged layer, and then obtained a continuous whole-brain dataset with a voxel resolution of 0.2 × 0.2 × 1 μm. To acquire the high-resolution dataset equally, we embedded the samples within resin to maintain the fine structures, such as synapses, during sectioning and imaging. Herein, we chose Lowicryl HM20 resin owing to its better hydrophobicity than other resins (24).

Because the fluorescent signals in the synaptic structure were weak, the preservation of the fluorescent intensity during sample processing and imaging was challenging. We modified the whole procedure for resin embedding. We found that the temperature during sample preparation affected the fluorescence intensity, especially for the weak signals. A lower polymerization temperature during resin embedding preserved more fluorescent signals and decreased the autofluorescence of tissues (25). To preserve the fluorescence intensity and increase the signal-to-noise ratio, we modified the embedding procedure with N, *N*-dimethyl-*p*-toluidine (DMT) as the accelerator for polymerization instead of the traditional accelerator. Then, we adjusted the parameters of the embedding process to decrease the polymerization temperature to −20 °C (*SI Appendix*, Fig. S3).

With the optimized resin and whole-brain imaging system, we acquired 3D datasets of the long-range projection and distribution of the presynaptic terminals of PV^+^ neurons. As shown in Fig. 1*I*, the axonal projections and presynaptic terminals of the PV^+^ neurons in the MS/VDB were reconstructed and registered to the Allen CCFv3 Brain Atlas (26). The axonal arbors were labeled with tdTomato, while synaptophysin was labeled with EGFP.

### Whole-Brain Distribution of Synaptic Terminals of PV^+^ Neurons in the Basal Forebrain.

**Fig. 2.**
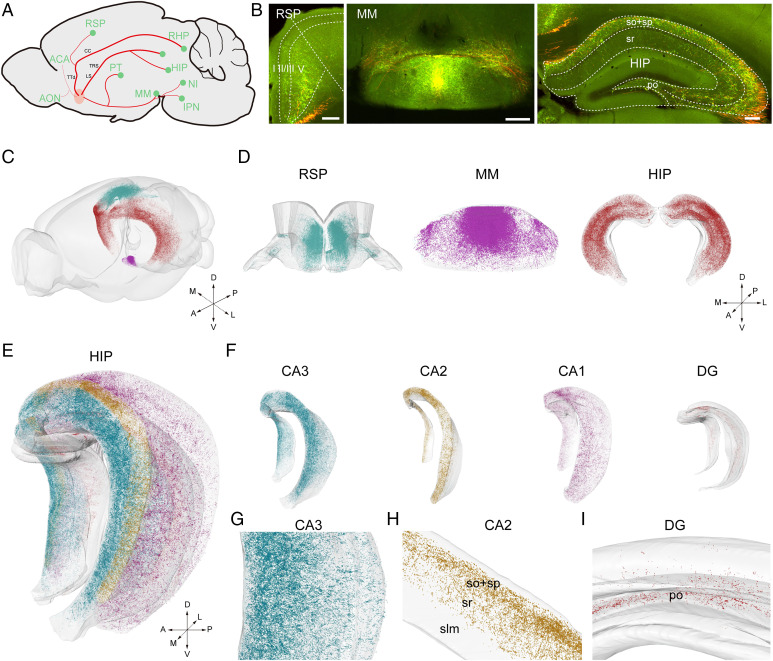
Visualization of the synaptic terminal distribution in the whole brain with fluorescence micro-optical sectioning tomography. (*A*) Schematics illustrating the major projection patterns of the PV^+^ neurons in the MS/VDB. (*B*) The PV^+^ neurons in the BF projected to special positions of the RSP, MM, and HIP. (Scale bar: 200 μm.) (*C* and *D*) Three-dimensional reconstruction of the RSP (blue dots), MM (magenta dots), and HIP (red dots). (*E* and *F*) HIP divided into CA1 (magenta dots), CA2 (yellow dots), CA3 (blue dots), and the DG (red dots). (*G–I*) Enlarged images of CA3 (blue dots), CA2 (yellow dots), and the DG (red dots). po, polymorph layer; slm, stratum lacunosum-moleculare.

The PV^+^ neurons sent abundant fibers and synaptic terminals to multiple regions, from the olfactory nucleus to the pons (*SI Appendix*, Fig. S4). From the reconstructed 3D data of the whole brain, we found that PV^+^ neurons governed their axonal projections via four separated trajectories. As summarized in Fig. 2*A*, one trajectory ascended to the frontal cortex and olfactory nucleus; this trajectory passed through the ventral tenia tecta to the olfactory nucleus and orbital cortex or through the dorsal tenia tecta to the infralimbic area (ILA) and prelimbic area (PL). The second trajectory was an ascending path that projected to the anterior cingulate area (ACA), retrosplenial area (RSP), motor cortex, and other isocortex areas. The third trajectory went through the MS, lateral septal nucleus (LS), and triangular nucleus of septum (TRS) and then mainly targeted the subregions of the hippocampal region (HIP), including Ammon’s horn (CA) and dentate gyrus (DG), while many fibers passed through the fimbria to the retrohippocampal region (RHP), including the parasubiculum (PAR), postsubiculum (POST), presubiculum (PRE), and subiculum (SUB). The fourth path was a descending trajectory, which passed through the tract and subregions of the hypothalamus under the anterior commissure and then extended to the amygdala, thalamus, midbrain, and pons. In the thalamus, the axonal fibers converged in the middle part of the thalamus and then projected anteriorly and dorsally to the habenula, while some fibers projected posteriorly to the pons and midbrain.

In addition, even though there were many axonal fibers in the TRS and ventral tegmental area (VTA), the synaptic density was low, which suggests that most of these axons were passing through rather than targeting these areas (*SI Appendix*, Fig. S5). In other regions, such as the periaqueductal gray (PAG), pontine gray (PG), tegmental reticular nucleus (TRN), medial amygdalar nucleus (MEA) and cortical amygdalar area (COA), the axons were sparse, but the synapses on individual axons, which should be the targeted terminal, were dense. Furthermore, with the merged images of the axonal arbors and SypEGFP, we investigated the synaptic location in these brain regions. As shown in *SI Appendix*, Fig. S6*A*, SypEGFP was distributed on some axons and gathered in some of them. Similar to the distribution patterns of axons, the synaptic location also showed subregion preference and gathered in different patterns. In the HIP, some SypEGFP presented as a cluster that may be around a neuron body, while there was no synapse on some axons (*SI Appendix*, Fig. S6*B*).

To further analyze the long-range distribution patterns of the PV^+^ neurons in targeted areas, we detected the SypEGFP signals in the whole-brain datasets and compared them among different brain regions through 3D reconstruction. We recognized and extracted the signals using the Farsight algorithm (27). With the precise 3D dataset, we could reconstruct each brain region according to the reference atlas and locate SypEGFP in the anatomical position. Even in different parts of individual regions, the SypEGFP distribution was not equal (Fig. 2 *B*–*D*). These PV^+^ neuron projections from the BF showed subregion preferences in the cortex, HIP, and hypothalamus. In the cortical areas, these PV^+^ axons mainly projected to the RSP and ACA with the layer-preferred pattern that the axons were distributed in all layers, except layer 1, and gathered in deep layers, especially in layer 5 (Fig. 2 *B* and *D*), while a few PV^+^ axons were in the motor cortex. In the medial mammillary nucleus (MM) of the hypothalamus, more SypEGFP located in the dorsal-medial part, with little SypEGFP in the lateral part (Fig. 2 *B* and *D*). From the 3D view, we found that SypEGFP were located more in the anterolateral parts of the HIP than in the posteromedial parts (Fig. 2 *E* and *F*). We also observed this phenomenon in CA3 (Fig. 2*G*). Furthermore, we analyzed the distribution of SypEGFP in different subregions of the HIP. As shown in Fig. 2 *B* and *H*, SypEGFP showed a preferential location in CA1 and CA2, with more SypEGFP located in the upper layers, including the stratum oriens (so), pyramidal layer (sp), and stratum radiatum (sr). Moreover, we also found that PV^+^ neurons preferentially innervated the polymorph layer of the DG (Fig. 2 *B* and *I*).

### Quantitative Analysis of Synaptic Terminals of PV^+^ Neurons in the Whole Brain.

**Fig. 3.**
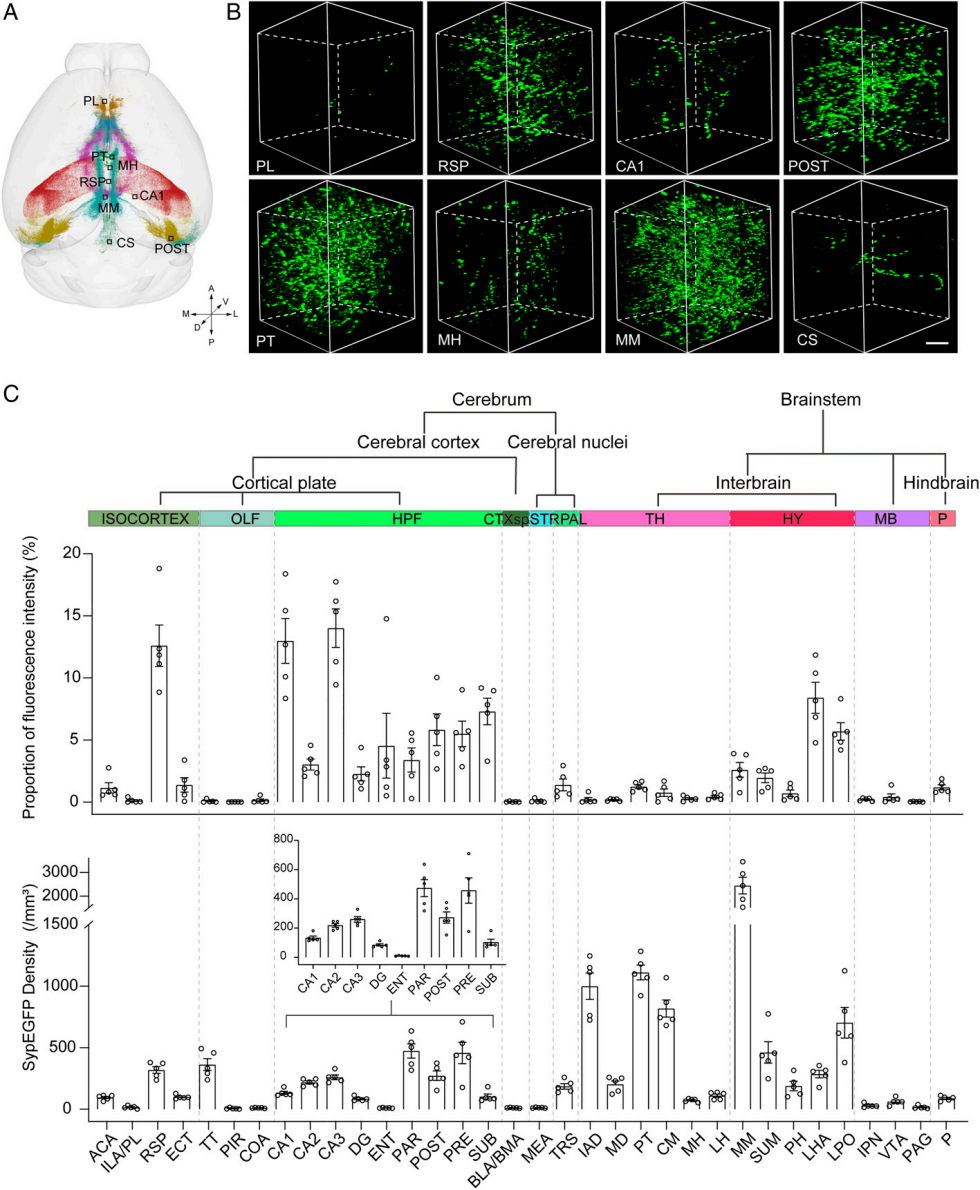
Quantitative analysis of synaptic terminals in the targeted brain regions. (*A*) Three-dimensional reconstruction of the whole brain. Different colors represented different nuclei. (*B*) The data block shows the distribution of GFP^+^ dots in different brain regions from (*A*). Block size: 100 × 100 × 100 μm. (Scale bar: 20 μm.) (*C*) The proportion of fluorescence intensity in 34 subregions (intensity, mean gray value per punctum [AU.]) (*n* = 5). Details of the proportions of fluorescence intensity in HPF subregions. Synapse numbers per unit volume in 34 brain subregions (average number synapse of 1 mm^3^) (*n* = 5). A, anterior; D, dorsal; L, lateral; M, medial; P, posterior; V, ventral; BLA, basolateral amygdalar nucleus; BMA, basomedial amygdalar nucleus; CS, superior central nucleus raphe; ECT, ectorhinal area; HY, hypothalamus; IPN, interpeduncular nucleus; LPO, lateral preoptic area; MB, midbrain; OLF, olfactory areas; STR, striatum; PAL, pallidum; TH, thalamus; TT, taenia tecta.

To quantitatively analyze the distribution patterns of synaptic terminals in the whole brain, we employed three parameters for the quantitative analysis of the synaptic terminals in 34 brain regions (Fig. 3 *A* and *B*). The proportion of fluorescence intensity reflected the distribution of synaptic terminal preference in different downstream regions, the number of synapses connected to a single soma per unit volume reflected the connection strength of the region, and the average distance reflected the degree of SypEGFP aggregation. We analyzed the SypEGFP distribution throughout the whole brain (*SI Appendix*, Fig. S7*A*). In the region between Bregma −2 mm and Bregma −5 mm, most of these positive signals were located in the hippocampal formation (HPF) area. In the range between Bregma 0 and Bregma −1 mm, most of the positive signals were located in some subregions of the hypothalamus and thalamus, such as parataenial nucleus (PT). These results confirmed that the PV^+^ neurons in the MS/VDB mainly targeted the hypothalamus, thalamus, and HPF.

To explore the connection among these targeted regions of PV^+^ neurons in the BF, we generated a similarity matrix of the 10 main downstream regions (*SI Appendix*, Fig. S7*B*). The results showed that the isocortex, olfactory, HPF, and pallidum regions were clustered separately, while the cortical subplate (CTXsp) and striatum, thalamus and hypothalamus, and midbrain and pons could be clustered together. The different correlation clusters indicated distinct collateral projection patterns of the PV^+^ neurons in the MS/VDB to downstream brain areas.

To compare the preferential distribution of SypEGFP in downstream regions, we quantified the proportion of fluorescence intensity in each targeted region (Fig. 3*C*). We considered the fluorescence value of all brain areas as 100% and calculated the percentage of each brain area. The results showed that the percentage of HPF occupied more than half of all brain regions with synaptic terminals, while most of SypEGFP gathered in the CA1 and CA3 (12.98 ± 1.80% and 14.00 ± 1.56%, respectively). A small amount distributed in the CA2, DG, and entorhinal area (ENT) (3.02 ± 0.45%, 2.27 ± 0.57%, and 4.54 ± 2.61%, respectively) (Fig. 3*C*). This means that although the HPF was the main targeted area, some subregions in the HPF were areas that fibers passed through instead of the targeted area, as CA3, POST, and PAR received more PV^+^ neuron inputs than other subregions (CA2 and the SUB). In addition, there were abundant labeled puncta in the cortical areas that were concentrated in the RSP (12.59 ± 1.66%).

The SypEGFP in the hypothalamus was mainly distributed in the anterolateral regions, including the lateral hypothalamic area (LHA) and lateral preoptic area (LPO), and the posteromedial regions, including the supramammillary nucleus (SUM), MM, and posterior hypothalamic nucleus (PH). These circuits may play important roles in the function of these targeted regions that are involved in sleep modulation, learning, and memory.

Furthermore, we investigated the region connection strength of individual PV^+^ neurons. The number of positive cells in the injection site has a major influence on synaptic terminals in downstream regions. To eliminate the effect of differences in the numbers of starter cells among different samples, we counted the number of positive neurons. The number of somas of five mice was between 1,519 and 2,351, and we calculated the SypEGFP density using the following formula: SypEGFP density = number of SypEGFP/volume/number of positive neurons. The SypEGFP density in the targeted area was normalized to the number of labeled PV^+^ neurons, which reflected the connection strength of individual neurons in the specific downstream region. Among all targeted brain areas, the MM, which is important for spatial memory formation, was the region with the highest SypEGFP density (2,449.69 ± 350.98/mm^3^) (28). The piriform area (PIR), the main targeted area in the olfactory bulb, was the region with the lowest number of PV^+^ neuron projections (6.01 ± 2.28/mm^3^) (Fig. 3*C*). These results indicated that the projections from individual PV^+^ neurons differed in strengths in different targeted areas. For the HPF, although it received the most input from the BF, its number of inputs from individual PV^+^ neurons were lower than that of the thalamus, which means that more PV^+^ neurons projected to the HPF than to the thalamus.

In the hypothalamus, the LHA, LPO, and MM were the main innervated areas. In the subregions of the thalamus, the synaptic density of the interanterodorsal nucleus of the thalamus (IAD), PT, and central medial nucleus of the thalamus (CM) were relatively higher than that of the mediodorsal nucleus of the thalamus (MD), medial habenula (MH), and lateral habenula (LH).

The SypEGFP density might be affected by the volume of brain regions. To better understand the connection strength of the region, we analyzed the average distance of SypEGFP to reflect the degree of SypEGFP aggregation (*SI Appendix*, Fig. S7*C*). Many brain regions, such as the midbrain and pons, followed a trend in which SypEGFP density was inversely related to the average distance. Interestingly, we found that SypEGFP density varied greatly in regions of thalamus, but the average distance of SypEGFP varied little in the corresponding region. The SypEGFP density of COA was similar to that of ENT, but the average distance of COA was higher than that of ENT, indicating that the SypEGFP distribution of COA was more extensive, while the distribution of ENT was more concentrated. In short, the strength of the connections was reflected by a variety of parameters.

To better understand the distribution of SypEGFP in the subregion, we quantitatively analyzed the SypEGFP distribution on the anterior-posterior (A*–*P), the medial-lateral (M*–*L), and the dorsal-ventral (D*–*V) (*SI Appendix*, Fig. S8). As shown in *SI Appendix*, Fig. S8 *A* and *B*, we found the results were consistent with the above results in that the SypEGFP of MM were located in the anterior-medial part. In the RSP, we found significant differences in distribution of SypEGFP on M*–*L. Most SypEGFP terminals gathered in layer 5 (*SI Appendix*, Fig. S8 *C* and *D*). However, the distribution of SypEGFP in PT had no impact on A*–*P, M*–*L, and D*–*V. This result indicated that the SypEGFP were evenly distributed in this region (*SI Appendix*, Fig. S8 *E* and *F*). Quantitative analysis of the distribution of SypEGFP in three dimensions could provide a better understanding that the connection strength between the labeled neurons and the regions analyzed.

### Synaptic Terminal Degeneration in AD Mice.

In AD patients and animal models, the BF showed a pathological loss of neurons (29) and defective circuit functions (30), but the pathological changes in the projections of specific neurons were not clear, especially the synaptic terminals in the downstream areas.

**Fig. 4.**
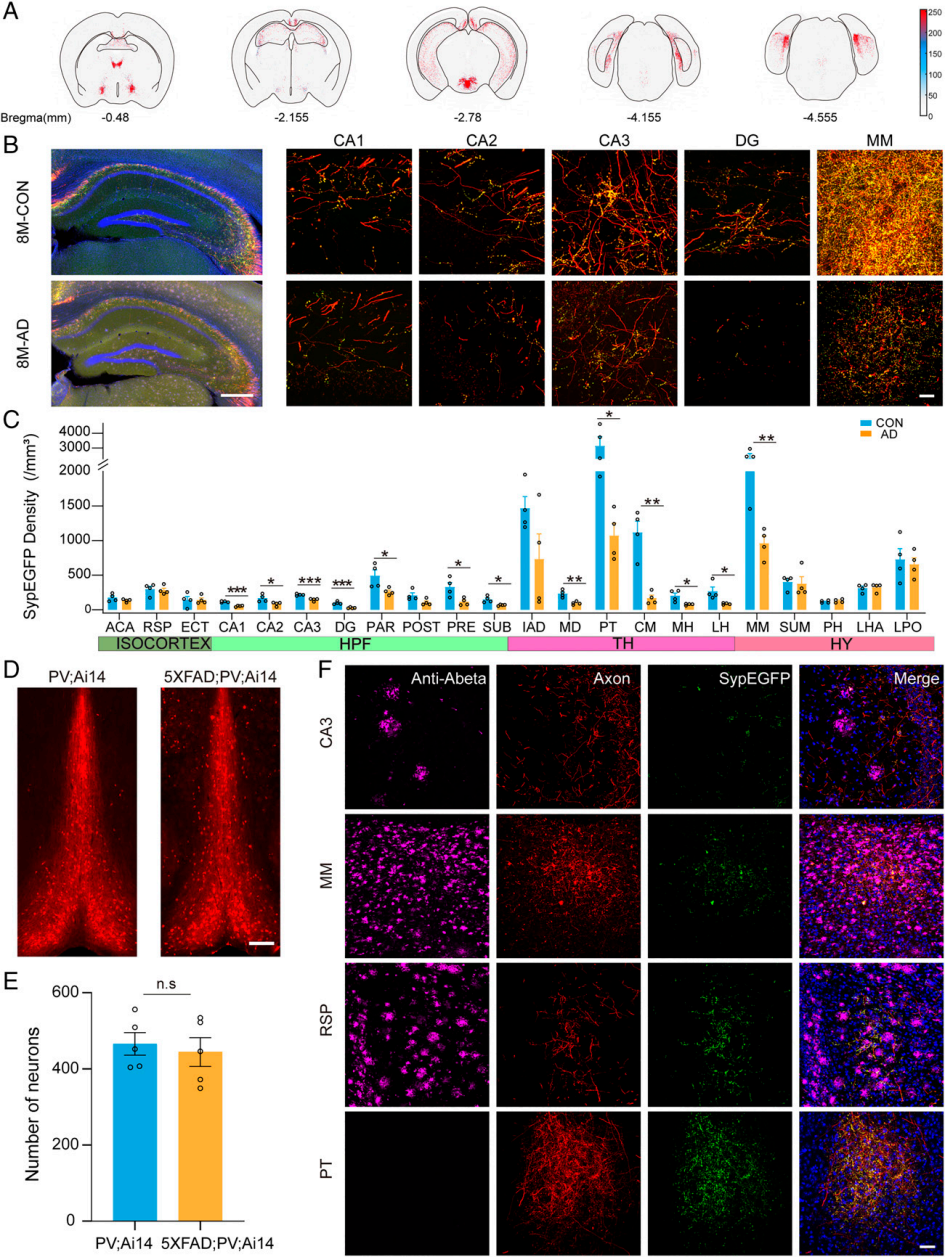
Synaptic degeneration of long-range projections in 5×FAD mice. (*A*) Synaptic distribution in different coronal sections. The red dots represent one synaptic terminal. (*B*) High-resolution images of the CA1, CA2, CA3, DG, and MM of PV-ires-Cre and 5×FAD; PV-ires-Cre mice. (Scale bar: 500 μm; 25 μm.) (*C*) Synaptic density comparison between CON and AD mice (*n* = 4, respectively) (multiple *t* tests followed by Holm-Sidak comparisons method, *P < 0.05, **P < 0.01, ***P < 0.001). (*D*) No major loss of PV neurons was observed in the MS/VDB of CON and AD mice. (Scale bar: 200 μm.) (*E*) Quantitative analysis of the number of PV neurons in the MS/VDB (*n* = 5, respectively). n.s means no significance. (*F*) Immunohistochemical staining of Aβ plaques in synaptic terminals brain regions (Scale bar: 50 μm.). ACA, anterior cingulate area; ECT, ectorhinal area; HY, hypothalamus; TH, thalamus.

We crossed PV-ires-Cre mice with 5×FAD mice and labeled the synaptic projections of PV^+^ neurons in the BF with viral tools. We selected the main targeted areas of the PV^+^ synaptic terminals in different coronal sections (Fig. 4*A*) and quantified the SypEGFP density in 22 brain regions (Fig. 4 *B* and *C*), which showed the regional connection strength of individual PV^+^ neurons. To compare the number of positive neurons labeled by viruses in CON (control group) and AD mice, we counted the neurons and found no significant difference. We already considered the number of positive cells when calculating SypEGFP density, which further eliminated the effect of different numbers of neurons at the injection sites. The HPF was a vulnerable area in AD mice, in which SypEGFP labeling in multiple subregions of the HPF decreased significantly, including CA1, DG, CA2, CA3, PAR, PRE, and SUB (*P* < 0.001, *P* < 0.001, *P* = 0.037, *P* < 0.001, *P* = 0.045, *P* = 0.026, and *P* = 0.012, respectively) (Fig. 4*C*). Apart from the HPF, the SypEGFP labeling in the thalamus was also reduced, especially in the MD, PT, CM, MH, and LH, all of which contribute to working memory and cognition (31–34). In the hypothalamus, there were no obvious changes except for in the MM (*P* = 0.006) (Fig. 4 *B* and *C*), a nucleus involved in the formation of spatial memories (28). In the isocortex, no obvious decrease in SypEGFP labeling was observed.

To confirm the correlation between SypEGFP loss and amyloid-β (Aβ) plaques in local areas and neuron soma changes, we quantified the number of PV^+^ neurons and stained Aβ plaques. We genetically labeled PV^+^ neurons by crossing PV-ires-Cre mice with Ai14 mice and subsequently crossed them with 5×FAD mice. The number of PV^+^ neurons in the BF of AD mice did not change significantly compared with that in the control group (Fig. 4 *D* and *E*). The survival of PV^+^ neurons in the BF was not dramatically impacted in 8-month-old 5×FAD mice. In addition, we stained the Aβ plaque, one of the signature protein complexes of AD, via immunofluorescent staining (Fig. 4*F*). During this period of the 5×FAD mice, there were many Aβ plaques with different sizes and densities in various regions of the isocortex, HPF, and hypothalamus, such as CA3, MM, and RSP.

To our surprise, there was no obvious SypEGFP loss in the isocortex, although the SypEGFP originating from the PV^+^ neurons in the BF was surrounded by dense Aβ plaques (Fig. 4*F*). In addition, no Aβ plaques were found in the PT, which was consistent with previous reports that few puncta were found in other hypothalamic regions (35). These results indicated that the mice expressed pathological characteristics with regional preference in this period. In general, our results suggested that the synapses but not the soma of PV^+^ neurons in the BF in AD mice were impaired and that the number of synapses in multiple targeted areas in the HPF, thalamus, and hypothalamus decreased significantly.

## Discussion

Using viral tracing, optimized whole-brain imaging technology, and big data processing, we developed a pipeline to quantitatively map the projection patterns of specific neurons with synaptic information at the whole-brain level. With the continuous dataset, we reconstructed the axonal projection and synaptic distribution of PV^+^ neurons in the BF in three dimensions. In addition, quantitative analysis of the synaptic terminals showed brain-region-specific degeneration in AD mice, while the changes in synaptic terminals were unrelated to local Aβ plaques in some brain regions.

The detection of precise structures, such as an individual synapse, throughout the whole mouse brain poses a complementary set of challenges for imaging. Although many precise imaging methods have been developed and used to analyze the synaptic distribution in brain slices or local areas and even to trace individual axons in tissue, the connection patterns at the synaptic level throughout the whole brain are still unknown. Sample processing is necessary to maintain the subcellular structure and fluorescent signals. However, the chemical treatment and changes in physical conditions during sample processing, such as the high polymerization temperature used in resin embedding and the chemical reagents used in tissue clearing methods, can easily decrease the fluorescence signal and increase the autofluorescence of biological tissues. These factors make it difficult to continuously detect the synaptic signals from the surface to the deep regions of the whole mouse brain equally, which limits the application of whole-tissue imaging, such as two-photon and light sheet systems, to comprehensively analyze weak signals and fine structures, such as to accurately quantify the distribution of synapses in the whole brain.

We confirmed the specificity of infected neurons in the injection site and the accuracy of synaptophysin localization via immunohistochemical staining. Quantification of the number and distribution of the somas in the injection site are particularly important in the experiment. Therefore, the viral titer, injection dose, and injection coordinates we used were consistent through the experiment. As shown in *SI Appendix*, Fig. S1*A*, the distribution of neurons at the injection sites were consistent in different samples.

Three parameters were employed to analyze the synaptic terminals in the targeted regions. First, the proportion of fluorescence intensity in each targeted region was used to show the distribution of synaptic terminal preference in different regions. Second, the SypEGFP density reflected the strength of the connections between the PV^+^ neurons and the targeted regions. Considering that the number of starting cells affected the SypEGFP density in the downstream region, we counted the number of positive neurons at the injection site and calculated the SypEGFP intensity of individual PV^+^ neurons (SypEGFP density = number of SypEGFP/volume/number of positive neurons). Finally, to better understand the connection strength, we calculated the average distance in each region. Consistent with previous reports (36), axons and synaptic terminals of PV^+^ neurons were found in the HPF, hypothalamus, and thalamus in the whole-brain distribution analysis, while the 3D reconstruction showed a subregion preference of synaptic terminals in the downstream brain regions. In the HIP, the terminals were mainly located in the so and sr layers in CA1 and the caudal side of CA3, while the synaptic density in the PAR, POST, and PRE were higher than that in the CA and DG. These projection preferences may play specific roles in specific functions. Moreover, we found that some SypEGFP accumulated in the basket form in the HIP, which has been shown in GABAergic and PV^+^ septohippocampal circuits (36). These circuits may form axosomatic synapses with hippocampal neurons to produce a greater change in membrane potential than the postsynaptic current generated by axodendritic contacts and therefore have a greater impact on action potential output. From the 3D reconstruction of whole-brain datasets, we found that some thalamic subregions, such as the IAD and CM, were innervated by PV^+^ neurons, which has not been reported in the previous literature. In the hypothalamus, synaptic terminals were mainly distributed in the MM and may play an important role in learning and memory. In addition, we calculated the average distance of SypEGFP to further reflect the distribution mode of synaptic terminals. The distribution of synaptic terminals in the COA is less but more dispersed, while it is less in the ENT but more concentrated. Different parameters reflect the connection strength between the BF or a single neuron and the targeted region from various perspectives.

Synaptic loss (37, 38) and axon degeneration (39, 40) in AD have been observed in both patients and animal models. 5×FAD mice have typical AD pathological features, including neuron loss, amyloid plaque accumulation, synapse degeneration, and neuroinflammation. Here, we discovered that the synapse loss of PV^+^ neurons was not universal but showed a brain-region-specific pattern, including a decrease in the HPF and some subregions of the thalamus and hypothalamus, which may be related to memory, such as the MM. However, no significant changes were found in other subregions, such as the LHA and LPO. These results were consistent with those of previous studies on the decrease in the GABAergic septohippocampal pathway in AD mice (41). Our similarity matrix showed that the synaptic projections in the HPF and hypothalamus were distinct (*SI Appendix*, Fig. S7*B*). Thus, it is very likely that different PV^+^ neurons innervated the HPF and hypothalamus. Together with the results of our previous study of axonopathy and Aβ plaques in the whole brain (35, 42), a pathway-specific manner of neuron degeneration was observed, in which specific neuron types were particularly vulnerable to AD progression. The results of the present study showed that the density of Aβ plaques in the MM was higher, while the number of PV^+^ synapses in the MM showed a greater decrease than that in CA3 (Fig. 4 *C* and *F*), which was consistent with the original assumptions that synapses degenerate, particularly near amyloid plaques (43). However, although a large number of Aβ plaques were observed in the isocortex, such as the RSP, the density of synapses did not change obviously. Moreover, there were many special regions, such as the PT and other subregions of the thalamus, that showed an obvious loss of synapses but no Aβ plaques (Fig. 4 *C* and *F*). These results indicated that synapse degeneration may be affected by local Aβ plaques in some brain regions but was caused by other factors in other regions, such as the thalamus. First, instead of Aβ plaques, soluble amyloid oligomers (44), which cannot be easily detected with staining, may affect the synapse structure in the local area. Second, the long-range PV^+^ axon projections to these targeted areas may pass through regions with heavy Aβ plaques and then suffer pathological injury in areas with high numbers of synaptic terminals. Third, our previous work showed that different BF neurons preferentially connect with different targeting circuits. The PV^+^ neurons in the BF that project to the isocortex and thalamus may degenerate in a special pattern. Therefore, the relationship between Aβ plaque accumulation and synapse loss may be highly complex, and further investigation is needed to elucidate this relationship.

With synaptic viral labeling, a modified whole-brain processing method, and a precise imaging system, we obtained continuous whole-brain datasets of the axonal and synaptic projections of PV^+^ neurons in the BF and identified the synaptic degeneration preference in the AD model. This work provides a way to investigate the projection patterns of specific neurons with synaptic information in the whole brain, which can be very helpful for understanding the structures and functions of neural circuits in both physiological and pathological conditions.

## Materials and Methods

### Animals.

C57BL/6J, PV-ires-Cre, 5×FAD, PV-ires-Cre;Ai14, and 5×FAD;PV-ires-Cre;Ai14 adult mice (2–8 months old) were used. C57BL/6J, PV-ires-Cre (stock No: 008069), 5×FAD (stock No: 034848), and Ai14 reporter (stock No: 007914) mice were purchased from Jackson Laboratory. We crossed PV-ires-Cre mice and Ai14 reporter mice to obtain PV-ires-Cre;Ai14 mice. 5×FAD mice were crossed with PV-ires-cre and PV-ires-Cre;Ai14, respectively, and progeny were genotyped by PCR. Three- to four-month-old male PV-ires-Cre mice were used for the experiment shown in Fig. 3. The female PV-ires-Cre, 5×FAD;PV-ires-Cre, PV-ires-Cre;Ai14, and 5×FAD;PV-ires-Cre;Ai14 mice at 8 months old were used for the experiment shown in Fig. 4. The male C57BL/6J mice at 2 months old were used for the experiment shown in *SI Appendix*, Fig. S1. Mice were housed on a 12-h light/dark cycle with food and water provided ad libitum. All animal experiments followed procedures approved by the Animal Ethics Committee of Huazhong University of Science and Technology.

### Virus Injections.

The mice were deeply anesthetized with a mixture of anesthetics (2% chloral hydrate and 10% urethane dissolved in 0.9% NaCl saline) according to their body weight (0.1 mL/10 g) (RWD Life Science Co., Ltd). The eyes of the mice were coated with ophthalmic ointment. After the skin was cut open, a small hole was made above the MS/VDB (AP [anterior-posterior]: 0.98 mm, ML [medial- lateral]: 0 mm, DV [dorsal-ventral]: −4.8 mm) using a skull drill. We injected 200 μL AAV2/9-hSyn-flex-tdTomato-T2A-synaptophysin-EGFP-WPRE-pA (1 × 10^13^ genome copies mL^−1^) virus into the MS/VDB at a flow rate of 50 nL/min. After the virus injection, the skin was sutured with medical sutures, and then the temperature of the mice was maintained at 37 °C using a heating pad. Finally, we returned the mice to the cage and waited 6–8 wk for maximal expression before imaging. The viral tool was purchased from Shanghai Taitool Bioscience Co., Ltd.

### Tissue Preparation.

Mice were anesthetized with a mixture of anesthetics and subsequently perfused with 50 mL of 0.01 M PBS (phosphate-buffered saline; Sigma-Aldrich, cat. no. P3813), followed by 100 mL of 4% paraformaldehyde (Sigma-Aldrich, cat. no. P6148). Second, the brains were excised and postfixed in 4% paraformaldehyde for 24 h in a 4 °C dark environment. After fixation, the brain was rinsed with 0.01 M PBS at 4 °C to replace paraformaldehyde.

### Immunostaining.

For immunostaining, brains were sliced into 50 μm coronal slices by a vibration microtome (Leica, VT1000 S). The brain slices were washed in PBS three times at room temperature, blocked with 5% (wt/vol) BSA (bovine serum albumin) containing 0.3% Triton-X-100 (vol/vol) in 0.01 M PBS for 1.5 h at room temperature and then incubated with primary antibodies for 24 h at 4 °C. The following primary antibodies were used: anti-PV (1:500, mouse, Millipore, MAB1572), anti-synaptophysin (1:200, mouse, Abcam, ab8049), anti-synapsin (1:500, rabbit, Abcam, ab254349), anti-SV2 (1:500, rabbit, Abcam, ab32942), anti-SNAP-25 (1:100, rabbit, Abcam, ab108990), anti-syntaxin (1:100, rabbit, Abcam, ab272736), anti-beta amyloid (1:800, mouse, Abcam, ab126649), and anti-GFP (1:500 rabbit, Abcam, ab290). Then, the sections were washed in PBS four times for 15 min and incubated with the corresponding secondary antibodies for 1.5 h at room temperature. All antibodies were diluted in the same block solution.

### Resin Embedding.

For the embedding test, the brains were sectioned into 70 μm coronal slices. The slices were dehydrated in a graded ethanol series (50, 75, 95, and 100% ethanol at 4 °C for 5 min each) and were penetrated in a graded HM20 resin series (50, 75, and 100% HM20 resin at 4 °C for 15 min each) and 100% HM20 for 6 h at 4 °C. Finally, these samples were embedded in polymerization solution containing 0.7% DMT at −20 °C for 24 h. For whole-brain embedding, the brains were dehydrated in a graded ethanol series (50, 75, 95, and 100% at 4 °C for 1 h each) and were penetrated in a graded resin series (50, 75, and 100% HM20 resin at 4 °C for 2 h each). Subsequently, the samples were impregnated in 100% HM20 solution for 2 d at 4 °C and embedded in 100% HM20 with 0.7% DMT at −20 °C for 48 h. The 100% HM20 solution included 62.3 g monomer, 14.9 g crosslinker, and 0.6 g BPO (benzoyl peroxide). The 50 and 75% HM20 (wt/wt) were prepared from ethanol and HM20.

### Imaging.

For PV neuron immunostaining, the sections were imaged using a confocal microscope (Leica, SP8) equipped with a 20× (NA 0.7) objective. For synaptic markers and GFP immunostaining, the sections were imaged at multiple focal planes using a confocal microscope (Zeiss, LSM710) equipped with a 63× (NA 1.46) oil immersion objective. The contrast images of confocal and super resolution were imaged with LSM710 and HIS-SIM (High Sensitivity Structured Illumination) (45), respectively. We first imaged brain slices with an LSM710 equipped with a 63× (NA 1.46) oil immersion objective and then imaged the same position with HIS-SIM. To better improve the resolution and contrast in reconstructed images, a sparse deconvolution algorithm was used, as described in a previous publication (46). HIS-SIM was provided by Guangzhou Computational Super-Resolution Biotech Co., Ltd. Images were taken to verify virus expression using a virtual slide scanning system (Olympus, VS120) equipped with a 10× (NA 0.45) objective. As shown in *SI Appendix*, Fig. S5, representative whole-section images of synaptophysin expression were taken through a confocal microscope (Zeiss, LSM710) equipped with a 40× (NA 1.43) oil immersion objective.

For precise whole-brain imaging, the mouse brains were embedded with the optimized HM20 method and imaged using an fMOST system with a voxel resolution of 0.2 × 0.2 × 1 μm. Briefly, the sample was moved by XYZ staging for imaging. When one stripe of image was finished, the stage moved a stripe-width distance in the y direction, and imaging was repeated in the x direction. The imaging process was repeated until the entire coronal section was acquired. After imaging, the surface layer was removed by a diamond knife. During imaging, the sample was immersed in 0.05 M Na_2_CO_3_ solution. The raw data were preprocessed on a computing server (72 cores, 2 GHz/core) and a graphical workstation (Dell, Round Rock, TX; T7920).

### Measurement of SypEGFP Parameters.

With the ROI manager module in ImageJ software, the fluorescence intensity in different brain regions was calculated. We considered the fluorescence value of all brain areas as 100% and calculated the percentage of each brain area.

For SypEGFP density, the “Spots” function of Imaris 9.0 (Bitplane AG, South Windsor, CT) was used to quantify the SypEGFP number. The spot size (puncta diameter) was set to 0.8 μm, and background subtraction was selected. The split-touching object was set as 1 μm. The software performed automatic detection, and then the threshold was manually adjusted (usually there was no more than 5%). The SypEGFP density was calculated using the following formula: SypEGFP density = number of SypEGFP/volume/number of positive neurons. The Spots function of Imaris 9.0 was used to quantify the average distance to the nearest nine spots within the same spot component, and multiple values were averaged to obtain the average distance of the entire brain region.

### 3D Visualization.

For the synaptophysin point distribution of the 3D reconstruction, we used the Farsight algorithm to identify the center points with a resolution of 0.2 × 0.2 × 1 μm. Then, these points were transformed into an SWC format and registered to the Allen CCFv3 Brain Atlas (26). All datasets and outlines of the whole brain were loaded in Amira software at the same time to obtain pictures of whole-brain volume rendering. For the display of local brain areas, we used the algorithm to extract the points belonging to the brain area separately and put them into the outline of the brain area.

### Statistics.

All statistical graphs were generated using GraphPad Prism 6.01. We conducted multiple *t* tests to compare the differences. The confidence level was set to 0.05 (*P* value), and all results were presented as the mean ± SEM.

## Supplementary Material

Supplementary File

## Data Availability

All study data were included in the study and *SI Appendix*. Data can be downloaded at http://atlas.brainsmatics.org/a/tian2209, (47).
