# Supplementary material for: Dissection of the long-range projections of specific neurons at the synaptic level in the whole mouse brain
Source: Proc Natl Acad Sci U S A. 2022 Sep 26;119(40):e2202536119. doi: 10.1073/pnas.2202536119 (PMC9546530; doi:10.1073/pnas.2202536119)
Supplement: Supplementary File [file pnas.2202536119.sapp.pdf]

Supplemental Figures

Supplemental Figure 1

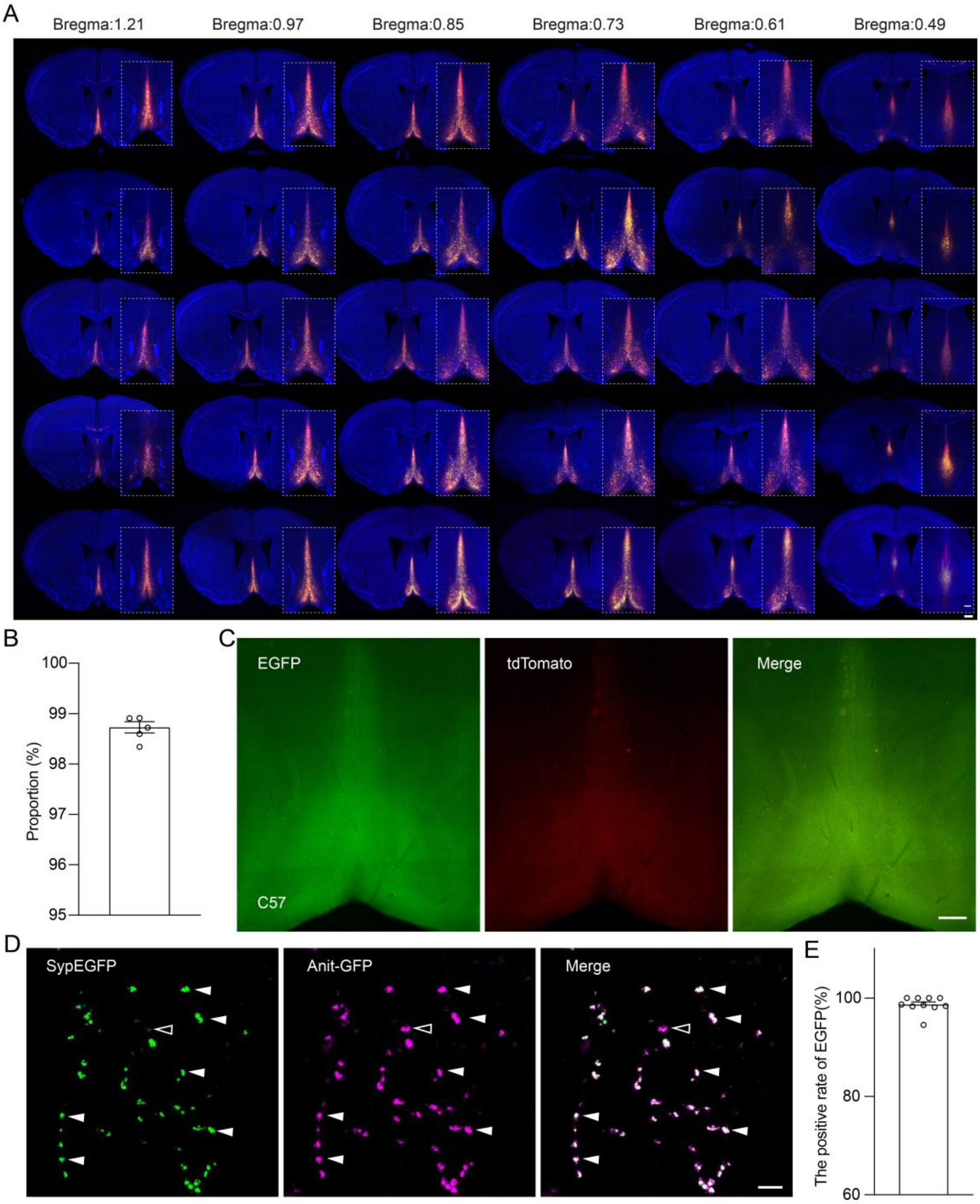

S1: Validation of virus labeling. (*A*) The coronal slices show that most of the labeled cells were restricted to the injection sites in different samples. Scale bar: 500 μm; inset: 200 μm. (*B*) The proportion of neurons in the injection site. (*C*) No neurons were labeled when the virus was injected into the C57 mouse brain. Scale bar: 100 μm. (*D*) Immunohistochemical staining and quantification of SypEGFP with an antibody against GFP. The arrows without a handle represent the expression of GFP, and the arrows with a handle indicate that the GFP signal is weak. Scale bar: 5 μm. (*E*)

Quantification analysis of the positive rate of EGFP.

Supplemental Figure 2

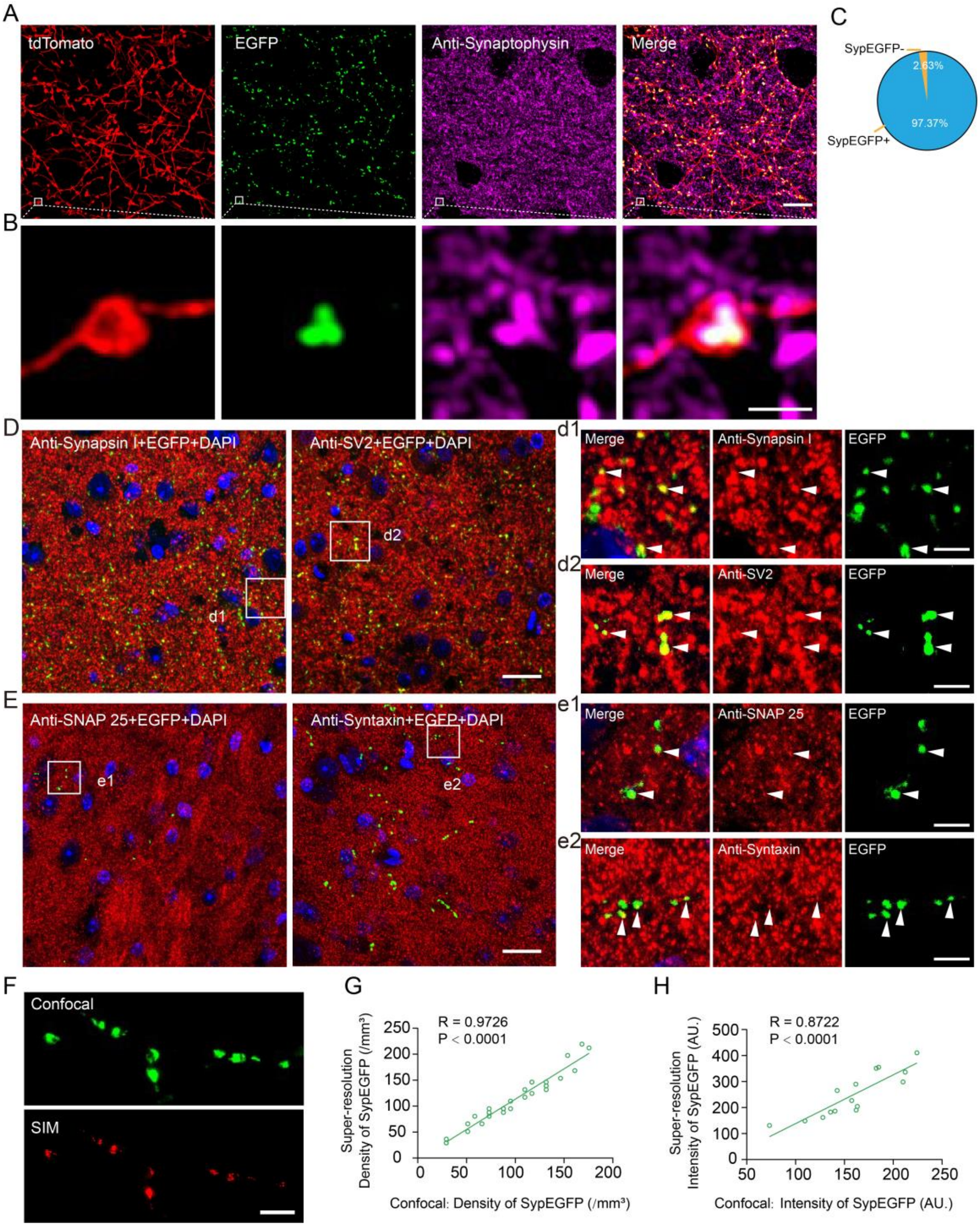

S2: Synaptophysin specificity verification and parameters analysis comparison. (*A*) Immunohistochemical staining of synaptophysin. Green: EGFP; Red: tdTomato; Magenta: Anti-Synaptophysin. Scale bar: 10 μm. (*B*) Enlarged images from A. Scale bar: 1 μm. (C) Quantification analysis of the positive rate of synaptophysin. (*D*) Immunohistochemical staining of synapsin and SV2. Green: EGFP; Red: anti-synapsin or anti-SV2; Blue: DAPI. Scale bar: 20 μm; d1 and d2: 5 μm. (*E*) Immunohistochemical staining against SNAP-25 and syntaxin. Green: EGFP; Red: anti-SNAP 25 or anti-syntaxin; Blue: DAPI. Scale bar: 20 μm; e1 and e2: 5 μm. (*F*) Image of single

fluorescent puncta with confocal microscopy and super-resolution microscopy. Scale bar: 5 μm. (*G*–*H*) High positive correlation of synaptophysin parameters quantified from confocal microscopy and super-resolution microscopy.

Supplemental Figure 3

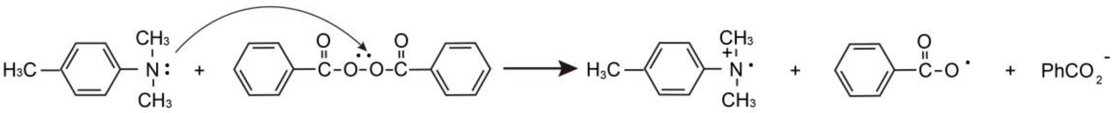

DMT BPO

S3: Principle of the redox reaction system. The main principle of this reaction is the reaction of DMT with BPO, which promotes the release of free radicals from BPO and then initiates the polymerization of monomers and crosslinkers.

Supplemental Figure 4

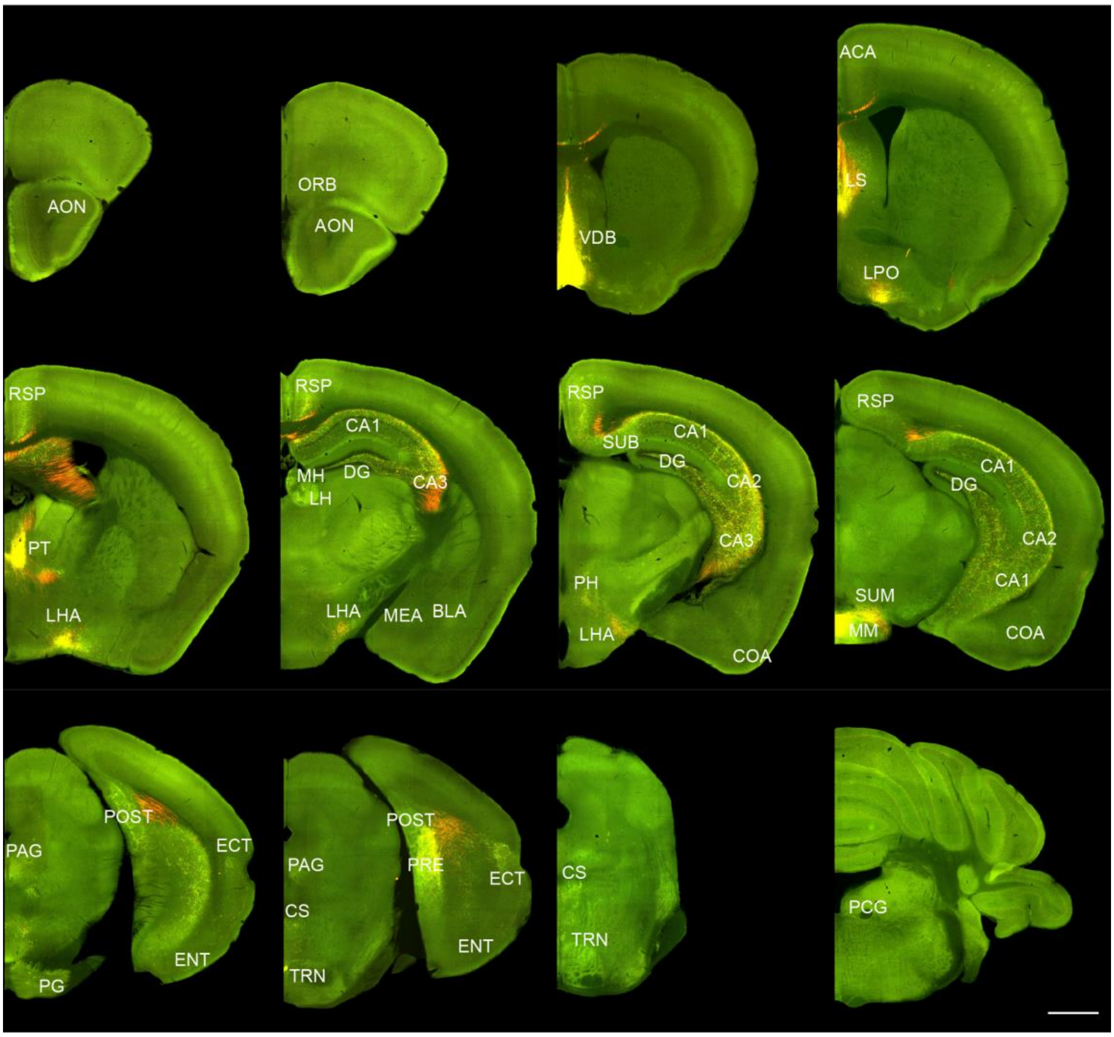

S4. Whole-brain imaging of dual-color-labeled PV-ires-Cre mouse brains. The projection was 70 μm. Scale bar: 1000 μm. The Allen Brain Atlas (Allen Institute, USA; http://atlas.brain-map.org/) was used as a reference for assigning nuclei or regions.

Supplemental Figure 5

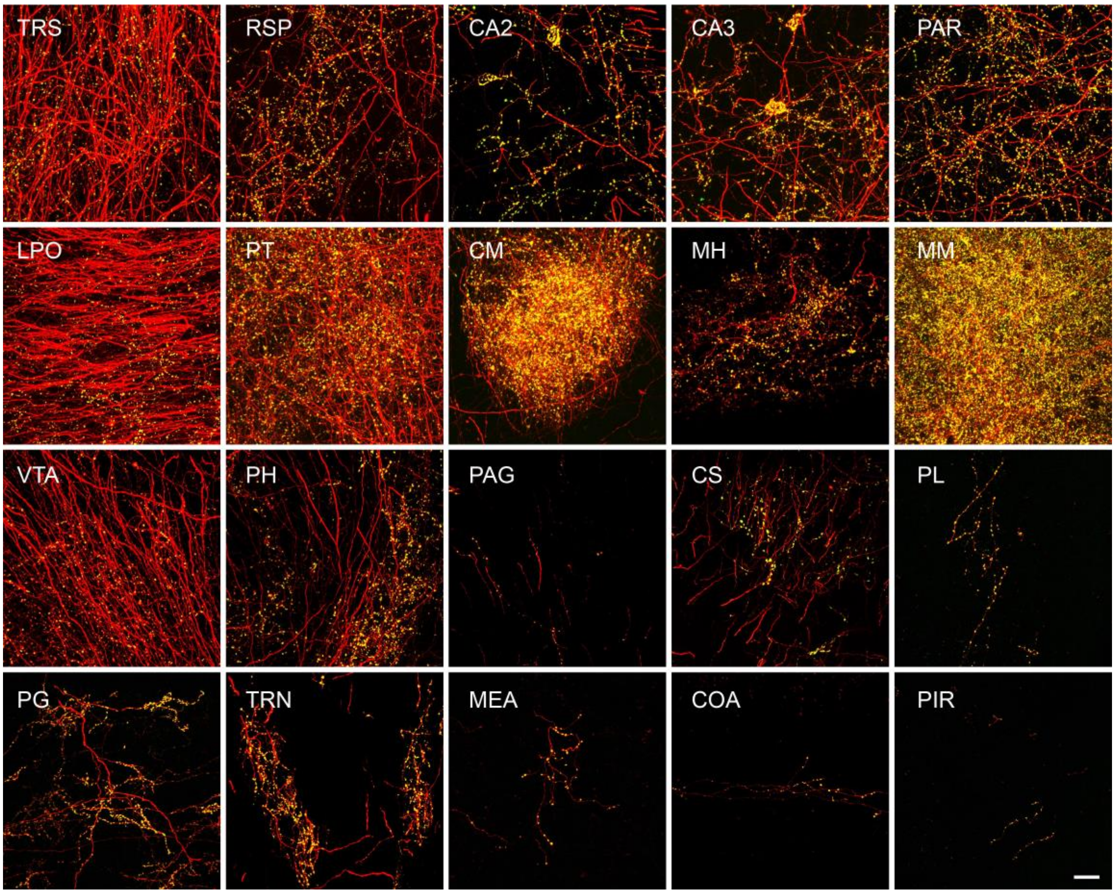

S5 Whole-brain distribution of axon and presynaptic terminals of PV^{+} neurons in the MS/VDB. Distribution of axons and presynaptic terminals in different brain regions. Scale bar: 25 µm.

Supplemental Figure 6

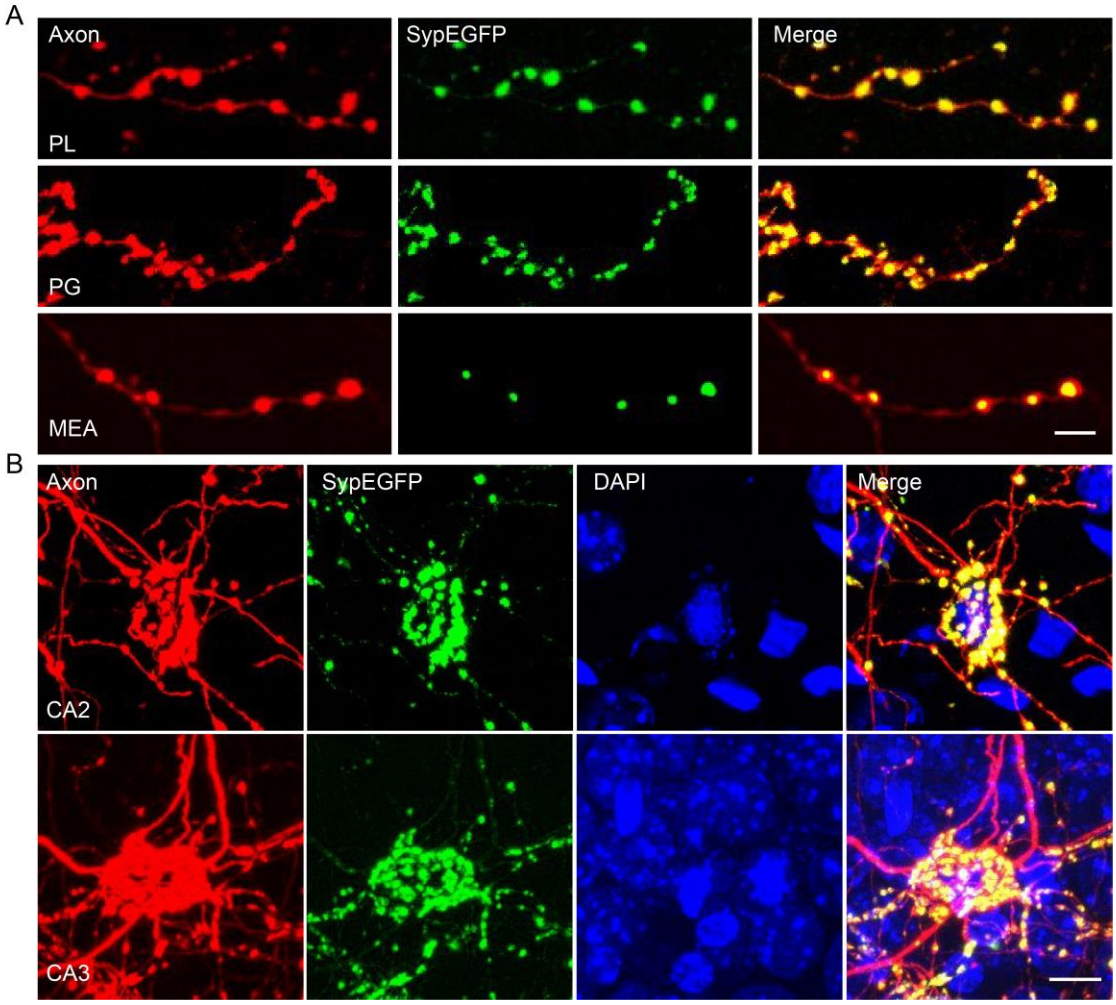

S6. Two patterns of synaptic connections. (*A*) The location of SypEGFP on the axons in the PL, PG, and MEA. Scale bar: 5 µm. (*B*) SypEGFP from clusters in CA2 or CA3. Scale bar: 10 µm.

Supplemental Figure 7

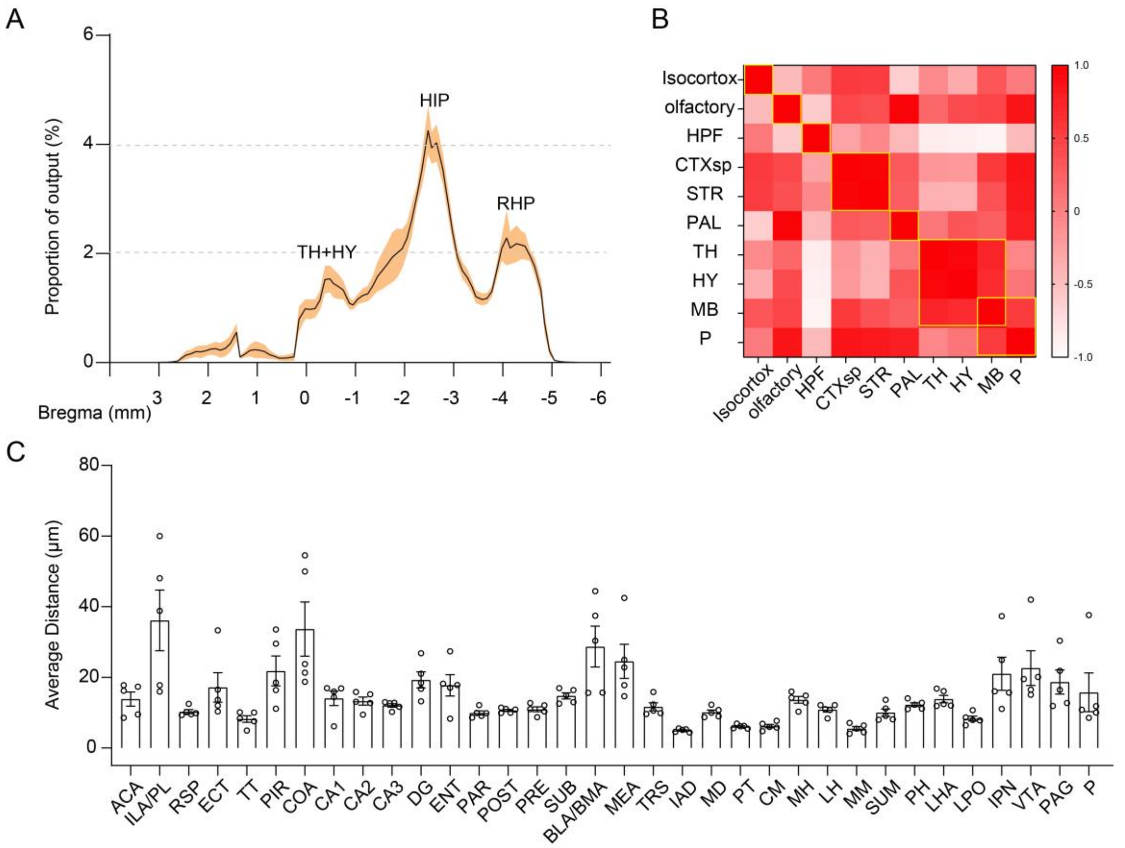

S7. Brain-region distribution of SypEGFP. (*A*) Proportions of SypEGFP in different coronal sections. (*B*) Similarity matrix between pairs of regions. (*C*) Average distance of SypEGFP in 34 brain subregions (μm) (n = 5).

Supplemental Figure 8

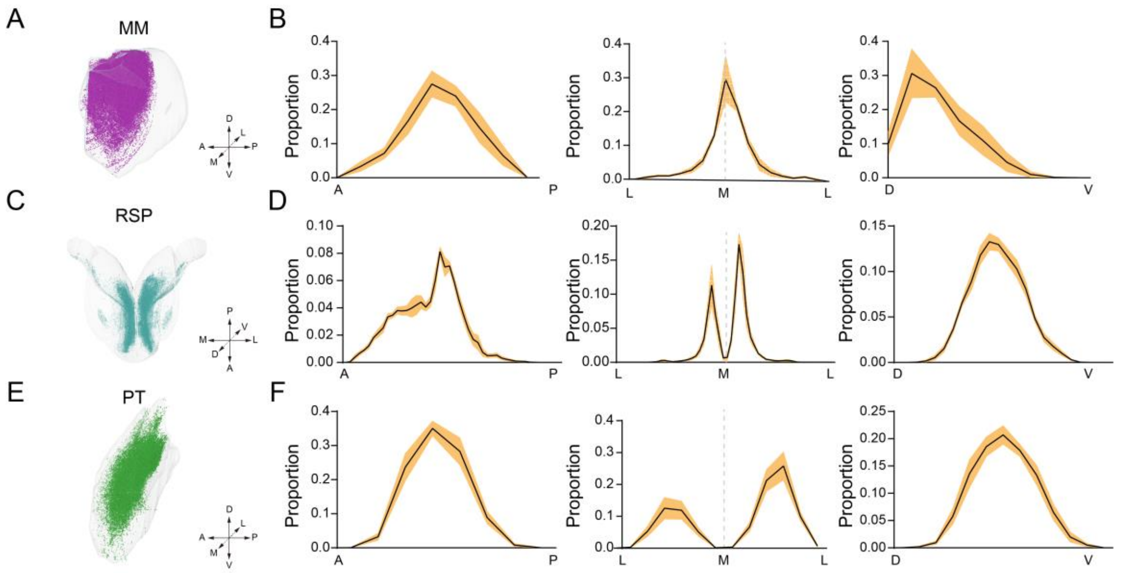

S8. Quantitative analysis distribution of the SypEGFP in the MM, RSP, and PT in three dimensions. (*A* and *B*) Distribution of SypEGFP on the A–P, M–L, and D–V regions in the MM. (*C* and *D*)

Distribution of SypEGFP on the A–P, M–L, and D–V regions in the RSP. (*E* and *F*) Distribution of SypEGFP on the A–P, M–L, and D–V regions in the PT.

Abbreviations

3D	three-dimensional
5×FAD	five familial AD mutations
ACA	Anterior cingulate area
AD	Alzheimer's disease
A-P	Anterior-Posterior
Aβ	amyloid-β plaque
BF	Basal forebrain
BLA	Basolateral amygdalar nucleus
BMA	Basomedial amygdalar nucleus
BPO	Benzoyl peroxide
CA	Ammon's horn
CA1	Field CA1
CA2	Field CA2
CA3	Field CA3
CM	Central medial nucleus of the thalamus
COA	Cortical amygdalar area
CTXsp	Cortical subplate
DG	Dentate gyrus
DMT	N, N-dimethyl-*p*-toluidine
D-V	Dorsal-Ventral
ECT	Ectorhinal area
ENT	Entorhinal area
fMOST	fluorescence micro-optical sectioning tomography system
GABA	γ-aminobutyric acid
HIP	Hippocampal region
HIS-SIM	High sensitivity structured Illumination
HPF	Hippocampal formation
HY	Hypothalamus
IAD	Interanterodorsal nucleus of the thalamus
ILA	Infralimbic area
IPN	Interpeduncular nucleus
ISO	Isocortex
LH	Lateral habenula
LHA	Lateral hypothalamic area
LPO	Lateral preoptic area
LS	lateral septal nucleus
MB	Midbrain
MD	Mediodorsal nucleus of thalamus

MEA	Medial amygdalar nucleus
MH	Medial habenula
M-L	Medial-Lateral
MM	Medial mammillary nucleus
MS	Medial septum nucleus
OLF	Olfactory areas
P	Pons
PAG	Periaqueductal gray
PAL	Pallidum
PAR	Parasubicolum
PG	Pontine gray
PH	Posterior hypothalamic nucleus
PIR	Piriform area
PL	Prelimibic area
POST	Postsubiculum
PRE	Presubiculum
PT	Parataenial nucleus
RHP	Retrohippocampal region
RSP	Retrosplenial area
so	Stratum oriens
sp	Pyramidal layer
sr	Stratum radiatum
STR	Striatum
SUB	Subiculum
SUM	Supramammillary nucleus
SypEGFP	synaptophysin-EGFP fusion protein
TH	Thalamus
TRN	Tegmental reticular nucleus
TRS	Triangular nucleus of septum
VDB	Vertical diagonal band
VTA	Ventral tegmental area
